# 78 Improving Delirium Communication and Documentation at the US Army Burn Center Burn Intensive Care Unit

**DOI:** 10.1093/jbcr/iraf019.078

**Published:** 2025-04-01

**Authors:** Jill Cancio, Kaitlin Reith

**Affiliations:** United States Army Institute of Surgical Research Burn Center; United States Army Institute of Surgical Research Burn Center

## Abstract

**Introduction:**

Delirium is a common occurrence in the intensive care unit (ICU). Up to 80% of ventilated ICU patients and 13% to 80% of burn patients develop delirium. However, up to 84.2% of delirium goes undiagnosed. Though burn patients are at increased risk of developing delirium, the literature on delirium assessment, prevention, and management in this population remains underrepresented. The purpose of this presentation is to describe findings from a performance improvement (PI) project intended to understand and improve rehabilitation and nursing documentation and communication regarding delirium in patients treated in the Burn ICU (BICU) at the a American Burn Association verified burn center in the southern region.

**Methods:**

FOCUS-PDCA PI model was utilized. Baseline were collected on all patients admitted to the BICU from 26Feb2023 to 08Mar2023 and compared to post-intervention data collected from 10Jun2024 to 21Jun2024. We selected the following metrics to evaluate program effectiveness: observation data via attending daily multidisciplinary rounds daily, review of the electronic medical record (EMR), and nursing and rehabilitation staff surveys. The plan for improvement included staff in-services, training and modification of electronic medical record templates, publication of a monthly newsletter, and one-on-one staff training. Nonparametric tests were used as indicated.

**Results:**

Data were collected on 15 patients admitted to the BICU within a 2-week period with a total of 457 observations at baseline and 14 patients with a total of 541 observations at the post-intervention timepoint. We found that 1) significantly more CAM-ICU scores were documented in the EMR by nursing per shift in the post-intervention period and reported on rounds (47% vs. 66% [p=0.028], and 4% vs. 47% [p< 0.01], respectively); 2) significantly more therapists understood types of delirium, delirium risk factors, and delirium interventions at the post-intervention timepoint (37% vs 100% [p< 0.01], 52% vs. 83% [p=0.026] and 85% vs. 100% [p=0.037], respectively), and 3) significantly more treatment modalities addressing delirium were documented in the occupational therapy treatment notes (4% vs. 36% [p< 0.001]).

**Conclusions:**

The project successfully enhanced communication, documentation, and staff understanding of delirium assessments and interventions at our Burn Center.

**Applicability of Research to Practice:**

This project marks the initial step towards establishing an interdisciplinary protocol for delirium prevention and management of delirium in the burn critical care environment.

**Funding for the Study:**

N/A

